# Fluorescence-Coupled Techniques for Determining Rose Bengal in Dermatological Formulations and Their Application to Ex Vivo Skin Deposition Studies

**DOI:** 10.3390/pharmaceutics15020408

**Published:** 2023-01-25

**Authors:** Qonita Kurnia Anjani, Sara Demartis, Fabiana Volpe-Zanutto, Huanhuan Li, Akmal Hidayat Bin Sabri, Elisabetta Gavini, Ryan F. Donnelly

**Affiliations:** 1 School of Pharmacy, Queen’s University Belfast, Medical Biology Centre, 97 Lisburn Road, Belfast BT9 7BL, UK; 2 Fakultas Farmasi, Universitas Megarezky, Jl. Antang Raya No. 43, Makassar 90234, Indonesia; 3 Department of Chemical, Physical, Mathematical and Natural Sciences, University of Sassari, Piazza Università 21, 07100 Sassari, Italy; 4 Department of Medicine, Surgery and Pharmacy, University of Sassari, Piazza Università 21, 07100 Sassari, Italy

**Keywords:** Rose Bengal, skin, fluorescence detection, method validation, ex vivo permeation tests, topical dosage forms, multiphoton microscopy

## Abstract

Rose Bengal (RB) is a fluorescent dye with several potential biomedical applications, particularly in dermatology. Due to RB’s poor physicochemical properties, several advanced delivery systems have been developed as a potential tool to promote its permeation across the skin. Nevertheless, no validated quantitative method to analyse RB within the skin is described in the literature. Considering RB exhibits a conjugated ring system, the current investigation proposes fluorescence-based techniques beneficial for qualitatively and quantitatively determining RB delivered to the skin. Notably, the development and validation of a fluorescence-coupled HPLC method to quantify RB within the skin matrix are herein described for the first time. The method was validated based on the ICH, FDA and EMA guidelines, and the validated parameters included specificity, linearity, LOD, LLOQ, accuracy and precision, and carry-over and dilution integrity. Finally, the method was applied to evaluate RB’s ex vivo permeation and deposition profiles when loaded into dermatological formulations. Concerning qualitative determination, multiphoton microscopy was used to track the RB distribution within the skin strata, and fluorescence emission spectra were investigated to evaluate RB’s behaviour when interacting with different environments. The analytical method proved specific, precise, accurate and sensitive to analyse RB in the skin. In addition, qualitative side-analytical techniques were revealed to play an essential role in evaluating the performance of RB’s dermatological formulation.

## 1. Introduction

Rose Bengal (RB) is a violet dye synthesised from fluorescein in the 19th century as a wool colourant. Chemically, RB, the 3′, 4′, 5′, 6′ -tetrachloro-2,4,5,7-tetraiodo-fluorescein is a highly water-soluble (100 g/L), fluorescent dianionic molecule belonging to the class of xanthene dyes [1]. Beyond its colouring abilities, RB exhibits intrinsic cytotoxicity towards different cancers [2,3,4,5,6] and microbial cells [7] and sono-photochemical properties, which allow its application in photodynamic (PDT) [8,9,10,11,12] and sonodynamic therapies (SDT) [13,14,15].

In recent decades, RB has garnered the scientific community’s interest, particularly in its potential role in managing dermatological diseases. Due to its intrinsic cytotoxicity, RB has been widely investigated as a potential chemotherapeutic in treating cutaneous melanoma, both early and metastatic [2,16,17]. To date, RB is undergoing clinical trials for the treatment of melanoma with the name of PV-10^®^ (Provectus Biopharmaceuticals, INC. Knoxville, TN, USA), which is a 10% *w*/*v* RB sterile and nonpyrogenic saline solution suitable for intralesional (IL) administration [18]. Despite its promising results, IL administration requires the healthcare assistance of professionals and causes significant pain and discomfort to the patient, which may result in poor compliance. More importantly, systemic phototoxicity following the intralesional administration of RB has been reported as a side effect [19]. Nevertheless, the IL route appears to be the most efficient administration considering RB’s high water-solubility and low permeability [1,15].

PH-10^®^ is a topical RB hydrogel formulation that selectively delivers RB to epithelial tissue and is currently involved in clinical trials [20]. PH-10^®^ has been employed for managing psoriasis and atopic dermatitis with encouraging results. Nevertheless, the action mechanism of RB in this disease has yet to be elucidated. Furthermore, RB alone or in combination with stimuli-responsive therapies has been reported to be a promising candidate for a plethora of therapeutic applications ranging from photochemical tissue bonding [20,21,22], white hair removal [23], plantar warts [24] and eradication of certain skin infections [7,25,26,27].

**Figure 1:**
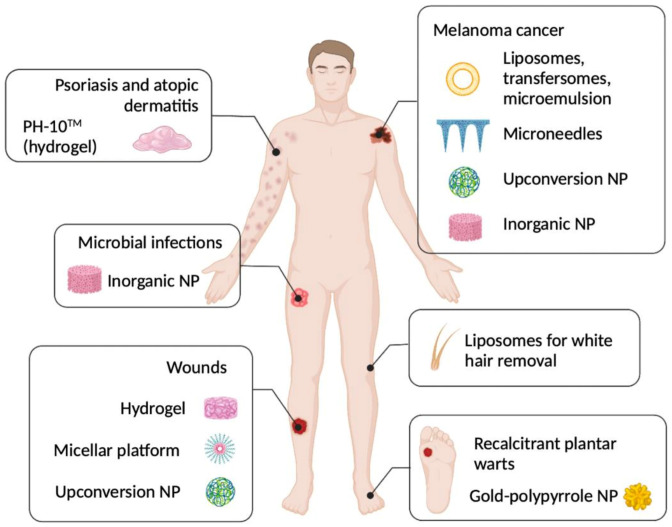
Summary of applications of RB-loaded formulations in managing dermatological diseases from the literature. NP: nanoparticles.

Research has been focused on developing advanced drug delivery systems that could help augment the efficacy of RB in dermatology (Figure 1) to obviate RB shortcomings, including low bioavailability and scarce permeation of biological membranes [1,28]. Some of these drug delivery systems include liposomes [29], transfersomes [30], micellar platform [31], microemulsion [32], hydrogel [33], upconversion particles [34,35], inorganic nanoparticles (NP) [36,37], hybrid NP [24] and dissolving polymeric microneedles [38].

Despite the role of RB in dermatology and the continuous investigation of advanced drug delivery systems, no validated quantitative method to analyse RB within the skin has been described in the literature. The detailed analysis of a drug within the skin strata is essential in estimating and understanding the permeation enhancement effect conferred by the delivery system used. RB is easily quantifiable using UV-visible and fluorescence techniques due to the presence of a conjugated ring system in its structure. The maximum wavelengths at which RB absorbs and emits have been determined in different solvents and are reported to be 546 nm and 567 nm in water, respectively [39]. To the best of our knowledge, only one validated RB quantitative method has been described, exploiting RP-HPLC with UV detection at 262 nm to quantify the analyte within eye surgical strips and not within the skin layers [40]. Other investigations mainly employed the UV-Vis spectrophotometer technique to determine RB content [31,41,42,43,44]. Many dermatological formulations contain biopolymers and lipids that must be removed before the analysis to mitigate any potential interference or matrix effect, which may interfere with RB quantification [45]. However, based on the chemical profile of RB, the complete isolation from excipients is not always successful, and some undesired traces can remain in the sample for analysis. RB strongly interacts with phospholipids, and its absorption and emission spectra are influenced by this interaction [30,46], as well as by the interaction with polymers [47] or skin components themselves [31]. In those cases, UV-Vis techniques may suffer from poor selectivity, sensitivity and relatively high inconstancy [39,48].

Fluorescence-coupled techniques have been widely used to evaluate the efficiency of drug delivery systems intended to be administered to the skin [49]; wide-field fluorescence and multiphoton microscopies are some of those techniques. Pena-Rodríguez and collaborators exploited the wide-field fluorescence microscopy to determine the biodistribution of Retinyl Palmitate-Loaded Transfersomes within the skin layers [49], aiming the epidermal delivery. Similarly, Mangion et al. [50] employed multiphoton microscopy to assess the follicular delivery of zinc pyrithione. Due to its intrinsic fluorescence, RB can be easily tracked within a biological milieu by means of the techniques mentioned above. In this regard, it has been reported that staining hepatoma cells with RB increased their visualisation under multiphoton microscopy compared with other dyes [51]. Fluorescence-based microscopies have been a helpful armamentarium for evaluating the dermatokinetic of topically applied RB [52]. Recently, we have exploited the multiphoton technique to estimate the penetration depth of an RB microneedle formulation into the full-thickness porcine skin [38].

Based on this rationale, this study intends to investigate the fluorescence-coupled techniques that should be considered to analyse RB in the skin, thus supporting the successful development of a skin-targeted delivery system. Two aims have been pursued in this work: (i) validating a selective and sensitive HPLC method for the RB quantitative determination in the skin matrix, and (ii) offering side-analyses techniques to qualitatively evaluate the performance of an RB formulation. Herein, we report, for the first time, the validation of an analytical method for RB quantification using the HPLC technique coupled with a fluorescence (FLD) detector. The validated analytical method was then applied to an ex vivo skin deposition experiment comparing three RB topical formulations (aqueous solution, cream and transfersome dispersion). The HPLC results were then cross-compared with multiphoton microscopy results. For this, the fluorescence spectra of skin spiked with RB and RB loaded in different formulations were acquired.

## 2. Materials and Methods

### 2.1. Materials

Rose Bengal disodium salt (RB), cholesterol, Span^®^ 80, ethanol, HPLC-grade methanol, ammonium acetate, sodium hydroxide, phosphate-buffered saline (PBS) tablets and dimethyl sulfoxide (DMSO) were purchased by Sigma-Aldrich (St. Louis, MO, USA). Aqueous cream BP was purchased under the brand name XBC aqueous cream (Buckinghamshire, UK). Ultrapure water was obtained from a water purification system (Elga PURELAB DV 25, Veolia Water Systems, Dublin, Ireland).

### 2.2. Instrumentation and Chromatographic Conditions

The apparatus employed to develop the proposed method was an Agilent Technologies 1220 Infinity compacted LC Series equipped with fluorescence (FLD) and UV-Vis detector with a binary pump, degasser and standard auto-injector set at room temperature (Agilent Technologies UK Ltd., Stockport, UK). The analyses of RB were performed on a C18 Phenomenex SphereClone^®^ column ODS(1) (150 × 4.6 m) with a particle size of 5 µm and pore size of 100 Å (Phenomenex, Cheshire, UK). All chromatograms were recorded and collected using the Agilent ChemStation^®^ Software B.02.01 (Santa Clara, CA, USA). For this study, we wanted to compare two standard analytical detectors to evaluate which detector conferred the most significant sensitivity. Upon identifying the most sensitive detector, this detector will be used to develop further and evaluate the analytical method for quantifying the molecule of interest, RB.

The mobile phase was a mixture of methanol and 20 mM ammonium acetate buffer solution at pH 8, used after degasification and filtration through membrane filters of mixed cellulose esters, 0.45 µm pore size and 47 mm diameter (GE Healthcare Life Sciences, Buckinghamshire, UK). The pH 8 buffer was obtained by accurately weighing and solubilising 1.54 g of ammonium acetate in ultrapure water in a 1000 mL volumetric flask. The pH was adjusted to 8 using NaOH. The isocratic elution of the mobile phase kept 60% methanol and 40% buffer of pH 8.0 with a flow rate of 1 mL/min. The excitation wavelength was set at 549 nm concerning UV-Vis detection. Regarding FLD detection, the excitation and emission wavelengths were set at 556 nm and 573 nm, respectively. The injection volume was 40 µL, and the run time was 7 min for UV-Vis detection and 6 min for FLD detection. The retention time was 3.1 min for UV-Vis and 4.1 min for FLD.

### 2.3. Standard Stock, Working Solution and Calibration Standards

To prepare the standard stock solution, 20 mg of RB were accurately weighed and dissolved in phosphate-buffered saline at pH 7.4 (PBS) into a 20 mL volumetric flask, reaching a 1 mg/mL concentration. One mL of the stock standard solution was withdrawn and further diluted up to 10 mL with PBS to obtain the standard working solution of 100 µg/mL final concentration. From the standard working solution, serial dilutions with PBS were performed to obtain seven calibration standards ranging from 1.33 to 80 µg/mL in the case of UV-Vis determination and from 0.16 to 10 µg/mL in the case of FLD determination.

### 2.4. Validation Process

The developed method was validated based on International Council on Harmonization (ICH) guideline, US Food and Drug Administration (FDA) guideline and the European Medicine Agency guideline [53,54,55] to ensure it fits the intended purpose.

#### 2.4.1. HPLC UV-Vis Method Validation

Concerning the HPLC method coupled with the UV-Vis detector, validated parameters were linearity, limits of detection (LOD) and quantitation (LOQ). Linearity was determined by evaluating the calibration curves from the calibration standards mentioned in Section 2.3. Six replicates of the calibration standards were analysed over three consecutive days (*n* = 6). The instrument response (peak area) was fitted as a function of the theoretical concentration, and the linear regression method was employed to determine the slope, y-intercept, and coefficient of determination (R^2^) using GraphPad Prism^®^ 9.4.1 (GraphPad Software, Boston, MA, USA). The LOD and LOQ were calculated from Equations (1) and (2), respectively, based on the standard deviation of the response and the slope from six calibration curves:

(1) $$\mathrm{LOD}=\frac{3.3\sigma }{S}\;,$$

(2) $$\mathrm{LOQ}=\frac{10\sigma }{S},$$

where σ is the standard deviation of the response and S is the slope of the calibration curve.

#### 2.4.2. HPLC-FLD Method Validation

Concerning HPLC-FLD determination, validated parameters were specificity, linearity, LOD and LOQ, inter-day and intra-day accuracy and precision.

#### Specificity

The specificity was investigated by comparing the chromatograms of six samples of blank PBS, blank samples from skin extract and PBS and skin samples spiked with a known amount of RB. The skin was prepared as reported below in Section 2.5.

#### Linearity, LOD and LLOQ

Linearity, LOD and LLOQ were calculated, as described above in Section 2.4.1., using the calibration standards mentioned in Section 2.3.

#### Inter-Day and Intra-Day Accuracy and Precision

Quality control (QC) solutions were prepared and analysed to calculate inter-day and intra-day accuracy and precision. Following the same procedure described in Section 2.3, three quality control (QC) solutions were prepared (1 µg/mL for low QC, 4 µg/mL for medium QC and 8 µg/mL for high QC). The inter-day accuracy and precision were determined by analysing the QC solutions within one run (*n* = 6), and intra-day accuracy and precision were observed between runs over three consecutive days (*n* = 6). The precision was expressed as the relative standard deviation (RSD%) of the responses of all samples (Equation (3)); the accuracy test indicated the relative error (RE%) between the experimental measurements and the theoretical concentrations (Equation (4)). The RSD% and RE% maximum values were set at 15%.

(3) $$\mathrm{RSD}\%=\frac{\mathrm{Standard}\;\mathrm{deviation}\;\mathrm{of}\;\mathrm{experimental}\;\mathrm{measurements}}{\mathrm{Mean}\;\mathrm{of}\;\mathrm{experimental}\;\mathrm{measurements}}\times 100$$

(4) $$\mathrm{and}\;\mathrm{RE}\%=\frac{\mathrm{Absolute}\;\mathrm{error}}{\mathrm{Theroetical}\;\mathrm{concentrations}}\times 100,$$

where the absolute error represents the deviation between the experimental measurements and theoretical concentrations.

#### Carry-Over

In order to evaluate the carry-over of RB, a QC sample at high concentration was injected, followed by the blank solution. The response of the blank solution obtained should not be more than 20% of the response from the sample at the LLOQ concentration [54,55].

#### Dilution Integrity

Dilution integrity was assessed by injecting the diluted samples (5 and 10 times) in PBS (pH 7.4) media. The accuracy and precision of obtained responses were then calculated [54,55].

### 2.5. Skin Extraction Recovery

**Figure 2:**
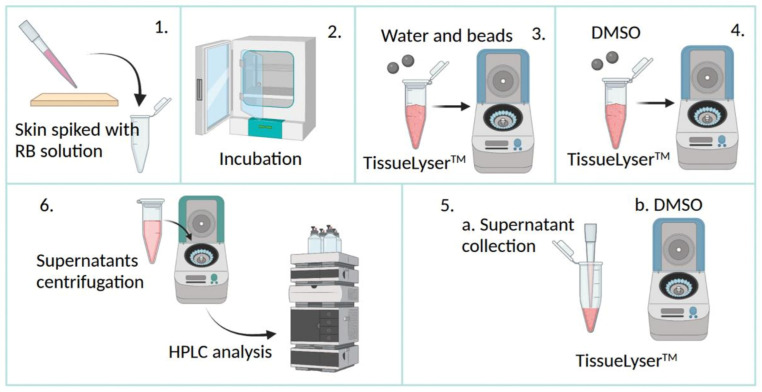
Schematic illustration of the skin extraction recovery process. RB: Rose Bengal; DMSO: dimethyl sulfoxide.

RB’s extraction recovery from dermatomed neonatal porcine skin was determined by analysing skin samples spiked with QC solution. The skin was obtained from stillborn piglets from Agri-Food and Bioscience Institute (Hillsborough, Northern Ireland, UK); the newborn piglets were instantly frozen at −20 °C and defrosted overnight before experimentation. First, full-thickness skin was excised using a surgical scalpel and cautiously shaved using a disposable razor (Gillette Blue II™, Gillette, Reading, UK). Then, dermatomed skin was isolated by trimming the shaved skin using an electric dermatome (Integra Padgett^®^ model B, Integra LifeSciences Corporation, Ratingen, Germany) to a thickness of around 350 µm. The skin was finally allowed to equilibrate for 30 min in PBS before the study. RB solutions were incubated with 100 mg of porcine skin in a 2 mL Eppendorf tube at 37 ± 0.5 °C for 24 h (Drying Oven VWRTM VENTI-LineTM VL 115, VWR International BVBA, Leuven, Belgium) to perform the skin extraction recovery test, as illustrated in Figure 2.

The skin samples were then cut into fragments by a scissor and lysed at 50 Hz for 15 min by a Qiagen TissueLyserTM LT (UK Quiagen Ltd., Manchester, UK), following the addition of deionised water (0.5 mL) and two stainless steel beads (diameter = 0.5 cm) (Qiagen, Hilden, Germany) [56,57]. Next, 1 mL of DMSO as extraction solvent was added [11], and samples were treated for a further 15 min (50 Hz); this step was repeated twice to ensure maximal RB extraction from the skin. The samples were then diluted in PBS, centrifuged at 14,000 rpm for 15 min (Sigma Laborzentrifugen GmbH, Osterode am Harz, Germany) to precipitate skin components, and the supernatants were meticulously collected and quantified by HPLC using the HPLC-FLD. Results were compared with those obtained from the control group consisting of the same weight of RB without skin, treated as above. The RB percentage skin extraction recovery (ER%) was expressed as the mean of the extraction recovery values obtained from the low, medium, and high RB amounts tested (Equation (5)):

(5) $$\mathrm{ER}\left(\%\right)=\frac{\mathrm{RB}\;\mathrm{quantified}\;\mathrm{in}\;\mathrm{skin}\;\mathrm{samples}}{\mathrm{RB}\;\mathrm{quantified}\;\mathrm{in}\;\mathrm{control}\;\mathrm{samples}}\times 100.$$

### 2.6. Preparation of RB Aqueous Solution, RB-Loaded Cream and RB-Loaded Transfersomes

RB was here loaded in three different formulations: (i) an aqueous solution, (ii) a water-based cream and (iii) a transfersomes dispersion.

RB aqueous solution (RB-S, RB = 2 mg/mL) was obtained by dissolving 10 mg of RB in 5 mL of ultrapure water. The solution was then vortexed for 30 s at 2500 rpm to ensure the complete solubilisation of the drug.

A water-based cream based on the British Pharmacopoeia (aqueous cream BP) was employed to obtain the RB-loaded cream (RB-C, RB = 0.2 mg/mg). RB (20 mg) was solubilised in 80 mg of the cream by a DAC 150 FVZ SpeedMixer (High Wycombe, England) at 3500 rpm for 5 min.

RB-loaded transfersome dispersion (RB-TF) (RB = 2 mg/mL) was already prepared and characterised by the same authorship [38]; the technique employed for the preparation was reverse-phase evaporation. Briefly, 20 mg of RB was solubilised in 10 mL of ultrapure water, and the lipid phase (142 mg of Lipoid S100, 26 mg of cholesterol, 14 µL of Span^®^ 80) was separately dissolved in 5 mL of ethanol. The two phases were subsequently submitted to a sonication process (60 s at 50% ultrasound (US) amplitude) using an ultrasonic probe device (Davidson & Hardy Ltd. cooperating with Fisher Scientific, Leicestershire, UK) to obtain a homogeneous dispersion. Afterwards, ethanol was evaporated by a rotary evaporator (Rotavapor, Buchi Labortechnik, Flawil, Switzerland) under a vacuum at 50 °C. The resultant formulation was conserved for 1 h at room temperature in dark conditions, and six additional sonication cycles (10 s of 50% US amplitude/20 s break) were finally applied.

### 2.7. Ex Vivo Skin Permeation and Deposition Study

An ex vivo permeation and deposition study were performed across dermatomed neonatal porcine skin, testing RB-S, RB-C and RB-TF. The skin was obtained and prepared as reported above in Section 2.5.

The study, conducted under infinite dose settings, employed Franz cells (Permergear, Hellertown, PA, USA) with a 1.77 cm^2^ orifice of an effective diffusion area of 0.36 cm^2^ and 12 mL receptor volume. The system’s temperature was thermally regulated at 37 ± 1 °C to provide a skin-surface temperature of 32 ± 1 °C at the skin surface [58]. First, the receiver compartment was filled with 12 mL of degassed PBS pH 7.4 as the receiver medium. Then, the skin was sandwiched between the donor and receiver compartment, with the subcutaneous side facing the receiver compartment. The skin was allowed to equilibrate for 30 min before experimentation [59].

**Figure 3:**
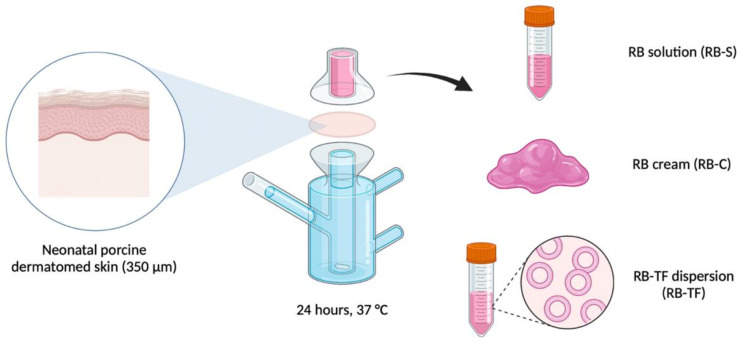
Schematic representation of the set-up employed for the ex vivo permeation and deposition study. RB-S: Rose Bengal solution, RB-C: Rose Bengal cream, and RB-TF: Rose Bengal transferosomes.

The three formulations were separately applied on the top of the skin surface: 100 µL of RB-S, 100 µL of RB-TF dispersion and 10 mg of RB-C. The donor and the sampling ports were sealed to minimise evaporation and contamination. The magnetic stirrers were set at 600 rpm to homogenise the receiver medium during the deposition study, which lasted 24 h (Figure 3).

Finally, both skin and receiver mediums were collected to quantify the RB amount deposited into the skin and the amount permeated. Any excess formulation from the solution, cream and dispersion on the skin surface was first wiped using wet tissue paper before processing the skin sample. The receiver medium was centrifuged, the supernatant was injected in the HPLC, and skin samples were processed as previously reported in Section 2.5 before the quantification analysis. The amount of RB permeated and deposited into the skin was estimated by referring to the calibration curve obtained by the HPLC-FLD method. To quantify the amount of RB deposited into the skin, the ER was considered as well, according to Equation (6):

(6) $$\mathrm{RB}\;\mathrm{deposited}\;\mathrm{in}\;\mathrm{the}\;\mathrm{skin}=\frac{\mathrm{RB}\;\mathrm{quantified}\;\mathrm{in}\;\mathrm{skin}\;\mathrm{samples}}{\mathrm{ER}}.$$

### 2.8. Multiphoton Microscopy Investigation

The deposition of RB-S, RB-C and RB-TF in the dermatomed skin was visually examined at the end of the ex vivo deposition study by multiphoton microscopy.

Micrographs were obtained using a Leica TCS SP8 multiphoton scanning microscope (Leica Microsystems Ltd., Milton Keynes, UK) set with an upright DM6 microscope body and a motorised stage. The Leica Application Suite X software (3.5.7.23225) was employed to process the images. Samples were excited with 549 nm laser lines from the Mai Tai Deep See Mode-Locked laser system (Newport-Spectra-Physics, Oxfordshire, UK). Fluorescence emission was detected via HyD GaAsP-spectral detectors for Rose Bengal dye between 557 nm and 660 nm. A water immersion objective HC FLUOTAR L 25× with 0.95 Numerical Aperture or dry objectives HC PL APO 10X/0.40NA or HC PL FLUOTAR L 40X0.60NA was employed to acquire images as suitable. Micrographs were acquired at a 1024 × 1024 pixel resolution format and a scanner speed of 400 Hz. Image analysis was performed using the Leica Application Suite X software (3.7.020979).

### 2.9. Examination of the Fluorescence Spectra

The fluorescence spectra of RB-S, RB-C, RB-TF, skin spiked with RB formulations and blank skin was carried out as previously reported by [46]. The spectra were recorded with a fluorescence spectrophotometer (Hitachi, Honshu, Japan), exciting each sample at 549 nm. Results were finally processed with GraphPad Prism^®^ 9.4.1.

In order to analyse RB-S, RB-C and RB-TF, the samples were diluted with PBS previously filtered (regenerated cellulose syringe filter, pore size 0.20 μm, filter size 15 mm; Albet LabScience, Dassel, Germany) to achieve a 10 µg/mL RB concentration. The auto zero was performed with blank PBS.

Regarding the analysis of skin spiked with RB-loaded formulations, the skin was first separately spiked with RB-S, RB-C and RB-TF and subsequently processed as reported in Section 2.5. Blank skin, submitted to the same treatment, was examined and used to perform the auto zero.

### 2.10. Statistical Analysis

Data were statistically analysed using GraphPad Prism^®^ 9.4.1 (GraphPad Software, San Diego, CA, USA). Multiple unpaired *t*-tests analysed accuracy, precision and skin extraction recovery. A two-way ANOVA was used to determine whether the RB permeated across and deposited into the skin. A *p*-value < 0.05 was used to indicate statistically significant differences in all cases.

## 3. Results and Discussion

### 3.1. HPLC Analytical Method

RB is a molecule with several applications in dermatology, and an analytical procedure to determine the molecule’s content within the skin upon delivery is highly demanded. The quantitative analysis of a drug in such a type of matrix is challenging because of the low expected concentrations, small sample volumes and the intrinsic structure of the skin itself. Indeed, the skin is considered a hard tissue, meaning a more robust extraction procedure and sample preparation are required to accomplish an accurate analysis relative to soft or tough tissues. Moreover, the endogenous components extracted from the biological matrix during sample preparation can easily interfere with the detection of the analyte. This is further complicated by the log P of RB (0.59), which causes the molecule to exhibit a natural affinity to skin components, making the extraction and, consequently, the quantification harder [60,61].

#### 3.1.1. Analytical Method Optimisation

The chromatographic conditions used as starting lines were those proposed by Mannan et al. to quantify RB from surgical strips [40]. This method was accurate, rapid and specific, but it was not sensitive enough for the purpose of our work, as it reported an LLOQ of 3 µg/mL and a LOD of 1 µg/mL. To improve the sensitivity, we first tried to change the excitation wavelength maintaining the UV-Vis detection. Secondly, we changed the technique of detection from UV-Vis to FLD. The three HPLC methods are summarised in Table 1.

Due to the extremely low water solubility of Rose Bengal (molecular formula C_20_H_4_Cl_4_I_4_O_5_), in this investigation, we employed the ionisable form of RB disodium salt (solubility in water: 100 g/L). Aqueous solubility is a desirable property for a drug molecule as it facilitates the interaction with the pharmacological target. For this purpose, the poor solubility of potentially active molecules can be modified by strategies recognised in contemporary medicinal chemistry, including exploiting any potential ionisable centres. The General Solubility Equation (7), used to predict the solubility for unionised molecules, can be modified to reflect the contribution of charges to solubility at any given pH by substituting logP for logD [62]:

(7) $$\mathrm{logS}=-\mathrm{logP}-0.01\times \left(\mathrm{Mpt}-25\right)+0.05$$

**Figure 4:**
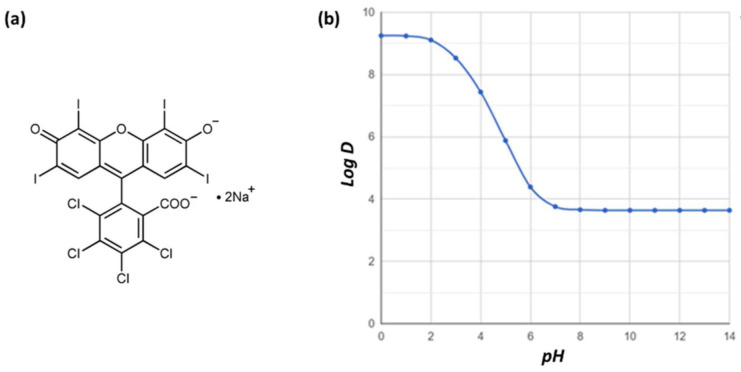
(**a**) Chemical structure of RB disodium salt. (**b**) LogD of RB as a function of pH was predicted using ChemAxon software (ChemAxon, Budapest, Hungary).

The increased ionisation reduces logD at given pH and increases the aqueous solubility. Since we used the ionisable form of RB, the mobile phase selection was pivotal to achieving an adequate ionisation degree [62]. The selected pH for the mobile phase was 8, as RB is entirely ionised and consequently solubilised (Figure 4b). The aqueous part of the mobile phase consisted of a phosphate buffer at pH 8.0, a value at which RB is entirely ionised, based on the logD prediction shown in Figure 4. 

**Figure 5:**
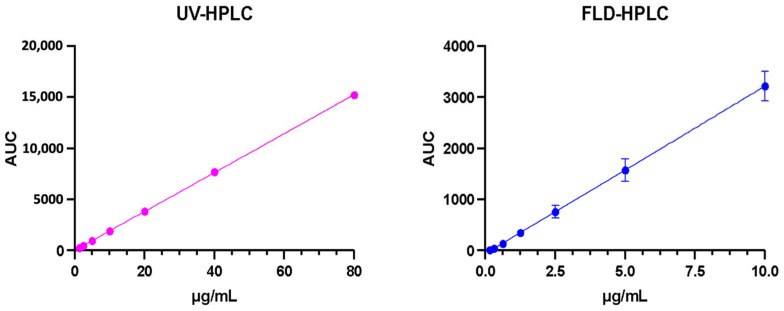
Calibration curves of RB obtained with UV-HPLC and FLD-HPLC detection (means ± SD, *n* = 6).

The starting method employed the excitation wavelength of 262 nm for RB determination in surgical strips [40]. Unfortunately, at lower wavelengths, the likelihood of interfering peaks arising in the chromatogram increases, especially within biological matrices [63]. Therefore, we decided to shift this value to the maximum RB absorption wavelength, set at 549–562 nm [39]. The two methods’ linearity, LOD and LLOQ, were calculated to identify the most suitable detector. Results are reported in Table 2, and the calibration curves are represented in Figure 5.

The data show that the calibration curves displayed a linear response with a regression coefficient (R^2^) ≥ 0.9999 over the concentration range evaluated. On the other hand, the LLOQ was found to be 0.54 µg/mL for the HPLC-FD method, which was more sensitive than the HPLC-UV method, which had an LLOQ of 1.83 µg/mL. The superior sensitivity obtained from HPLC-FD is attributed to the greater sensitivity conferred by fluorescent detectors compared with UV detectors [64]. Moreover, it is widely known that most polycyclic aromatic hydrocarbons, such as RB, can easily be observed via fluorescence-based techniques. The delocalised electrons in the aromatic rings can easily be excited. In addition, the stiff structure of polycyclic rings does not allow for efficient vibrational relaxation, enabling sufficient time for the emitted fluorescence to be detected [65]. Considering these outcomes, fluorescence detection was finally selected as the leader RB determination method and further evaluated.

#### 3.1.2. Specificity of the HPLC-FLD Method

**Figure 6:**
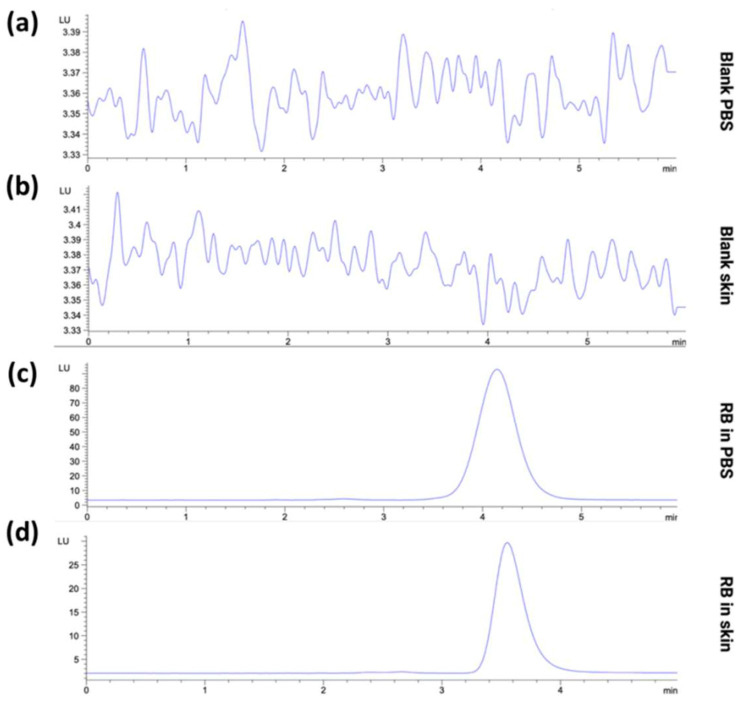
Representative HPLC-FD chromatogram of (**a**) blank PBS, (**b**) blank skin extract, (**c**) RB in PBS (10 µg/mL) and (**d**) RB from skin extract (5 µg/mL).

The specificity of the HPLC-FLD method was determined by evaluating the chromatograms of RB in the diluent and the presence of the skin matrix’s interferents. As reported in Figure 6, no interfering peaks were detected, suggesting the specificity of the current method. The fluorescent detector specificity prevents co-eluting peaks that typically arise when quantifying analytes from the biological matrix using HPLC-UV. Such advantage is attributed to the low concentration of naturally fluorescent compounds found within biological tissue. However, a change in the RB retention time in the presence of skin has been denoted, shifting from 4.1 min in PBS to 3.5 min in the skin. In this regard, it has already been described that the complex composition of the skin matrix provides samples that carry on a considerable diversity of endogenous substances, in contrast to the more homogeneous plasma samples [61]. This phenomenon, coupled with the known RB affinity for skin components, herein turned into a slight variation of the retention time without affecting the specificity of the method for the analyte.

#### 3.1.3. Accuracy and Precision of the FLD-HPLC Method

Accuracy and precision were evaluated over one day (intra-day variability) and three consecutive days (inter-day variability). The values are shown in Table 3. No statistically significant difference was observed between the theoretical and experimental concentrations (*p*-value > 0.05). The values of RE% for all QC samples were in the range of 1.17–2.42%. The precision, which indicates the method’s repeatability, exhibited an RSD% value from 0.98 to 3.06%, within the concentration range tested. The results confirmed that the new analytical method is accurate and precise since the values obtained were lower than the acceptable value of 15%; hence, they are under the recommendations for bioanalytical methods [60,66,67]. Based on these results, it can be assumed that the current approach is potentially appropriate for quantifying RB deposited into skin samples.

#### 3.1.4. Carry-Over

The signal of RB possibly interferes with the measurement of the blank solution; therefore, the carry-over evaluation was performed. In order to evaluate this, a high concentration of RB solution (50 μg/mL) was injected, followed by a blank sample. The result obtained was that the peak of RB was not detected in the sequence of blank samples. Accordingly, it indicates no carry-over effect in the developed HPLC method.

#### 3.1.5. Dilution Integrity

The dilution integrity was assessed by monitoring the RB concentration’s consistency upon diluting 5 and 10 times lower than the stock solution. As presented in Table 4, the results showed that the RSD% and RE% of both dilutions were in the range of 0.98–1.53% and 4.22–4.70%, respectively. This indicates that the dilution integrity is reliable for the developed HPLC method, as the values of RSD% and RE% were in the acceptable range, which is less than 15%.

### 3.2. Skin Extraction Recovery

A bead homogenisation approach has been employed to extract RB from the skin. This method breaks down the skin tissue by utilising stainless-steel grinding balls combined with a lysing solvent to extract RB. Through the high-velocity collision provided by a TissueLyser^®^, the beads grind and disrupt the cell membranes releasing the intracellular fluid [61]. The supernatant of the resultant homogeneous mix has been centrifuged and used to quantify RB. The bead homogenisation technique has already been applied for porcine tissues and suits all skin samples. Herein, dermatomed skin, with a thickness of 350 μm, was selected to limit the influence of the tissue thickness variability on the method’s validity [61,68]. It is important to remember that the extraction of RB was performed on the entire dermatomed skin without separating the epidermis from the dermis. Herein, DMSO was employed as lysing solvent, and it is recurrently used in biological studies because the solvent quickly penetrates and diffuses through biological membranes [69]. Furthermore, DMSO can dissolve the skin lipids or denature skin proteins. It is a very efficient solvent due to its intrinsic amphiphilic properties arising from its hydrophilic sulfoxide group and two hydrophobic methyl groups [70]. Considering the amphiphilic nature of RB, DMSO was considered the best candidate.

RB’s extraction recovery (ER%) was determined by comparing the concentration of different spiked skin samples to the standard solution as a control to understand if the technique mentioned above could provide reliable results. The results are displayed in Table 5. The RB recovered from dermatomed skin varied from 95.51 ± 2.49% to 97.62 ± 5.89%; the RSD% values fall within the range of 2.61–6.03%. No statistically significant difference between the RB concentration added and the concentration recovered was observed (*p*-value > 0.05). The recovery was within the 100 ± 10% limit reported in the OECD guideline, and RSD% values are below the maximum limit of 15%. These suggest that the extraction procedure was highly efficient, consistent, precise and reproducible [71,72,73].

### 3.3. Ex Vivo Skin Permeation and Deposition Study

The analytical and extraction methods were then used to quantify the amount of RB permeated into and across dermatomed porcine skin following a 24 h permeation study.

**Figure 7:**
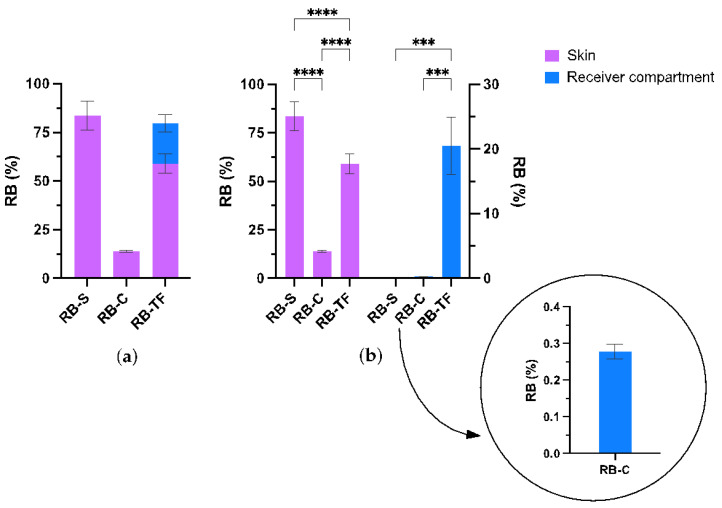
Amount of RB recovered after 24 h of an ex vivo deposition study across dermatomed porcine skin (*n* = 3). (**a**) Illustration of the total amount of RB detected at the end of the study. (**b**) Focus on the amount of RB detected within the skin layers and in the receptor compartment. **** *p*-value < 0.001, and *** *p*-value < 0.01.

The intact SC is the primary barrier to drug penetration [1]. To overcome this limitation, a drug that acts within the skin can be formulated in several topical delivery systems, from the most conventional to the most advanced. Conventional formulations include solutions, ointment, gels or creams, whereas newer drug delivery systems utilise nanoparticles and mechanical approaches that reversibly disrupt the SC [74,75,76]. Despite the range of approaches employed to enhance the delivery of therapeutics into the skin, nano-systems, such as liposomes, remain the most employed in clinics. With these assumptions, three types of topical formulations were tested herein: RB aqueous solution (RB-S), RB water-based cream (RB-C) and RB transfersome dispersion (RB-TF). Figure 7 reports the percentage of RB deposited within the skin and the receiver compartment.

The percentage of RB deposited into the skin was 83.57 ± 7.52 in the case of RB-S, 13.86 ± 0.48 in the case of RB-C and 59.07 ± 5.06 per RB-TF. At the same time, the percentage of RB permeated into the receiver compartment was 0.28 ± 0.02 and 20.53 ± 4.41 in the case of RB-C and RB-TF, respectively. In contrast, RB did not permeate across the skin when delivered by RB-S.

The reported data agrees with the characteristics of the prepared RB-loaded delivery system. The poor permeation profile for RB-S may be attributed to the physicochemical properties of the drug. The *stratum corneum* (SC) is the main barrier to the penetration of external agents. This is especially true for molecules exceeding 500 Da possessing anionic or cationic charges. RB disodium salt is a highly water-soluble amphiphilic drug with two anionic charges in the solution and a molecular weight of 1017.64 g/mol. RB aqueous solution (RB-S) did not permeate across the skin, and most of it was deposited into the skin tissue. In this investigation, no physical separation of epidermis and dermis was performed; however, RB is likely to accumulate in the epidermal layer without significantly reaching the dermis, as demonstrated in our previous work [30,38]. Incorporating RB into the aqueous cream (RB-C) led to the lowest deposition and permeation efficacy compared with RB-S and RB-TF. Aqueous creams are oil-in-water emulsions in which the water-soluble drug typically dissolves in the water phase [74]. The cream is composed of emulsifying ointment (30% *w*/*w*), soft white paraffin (15% *w*/*w*), liquid paraffin (6% *w*/*w*) and purified water, which is the typical composition for aqueous cream BP [77]. Considering RB’s affinity towards lipids, it is likely that RB may interact with the excipients present within the cream, cetostearyl alcohol and sodium lauryl sulphate, even post-application [46]. Aqueous cream BP is commonly used to manage dry skin by forming an oily layer on the top of the skin by decreasing the skin’s water loss via simple occlusion [78]. Based on this, we hypothesised that the oily phase of RB-C deposited on the top of the skin is responsible for the RB detected in the skin layers. This protective film, which serves as an occlusive layer, also mitigates the permeation of RB from the formulation into and across the skin following application.

Formulating RB into TF significantly improved the transdermal delivery of RB. RB-TF proved to be small unilamellar vesicles (SUV) with an average size of 62.91 ± 6.28 nm, a PDI of 0.271 ± 0.045, and a zeta potential of −38.47 ± 0.20 mV [38]. In this investigation, the amount of RB deposited into the skin layers following the application of RB-TF was considerably lower than RB-S (*p*-value < 0.001), and the amount permeating the whole skin was significantly higher than both RB-S and RB-C (*p*-value < 0.01). In this regard, TF is a liposome-like vesicle first developed by Gregor Cevc to improve the migration through the skin compared to its predecessors, the liposomes. In contrast to the rigidity of conventional liposomes, the presence of the surfactant within TF’s structure provides flexibility to the vesicle and allows TF to squeeze and pass-through skin pores without losing its integrity while being driven by the transdermal hydration gradient, ultimately improving the delivery efficiency [79].

These results remarked that the HPLC-FD method developed in this work could effectively quantify and elucidate the permeation profile RB across the skin from different delivery systems. The sensitivity conferred by the developed method would enable formulators to evaluate which formulation can exhibit the highest delivery efficiency. Such a decision would be paramount in moving the formulation into preclinical and clinical studies.

### 3.4. Multiphoton Microscopy Investigation

**Figure 8:**
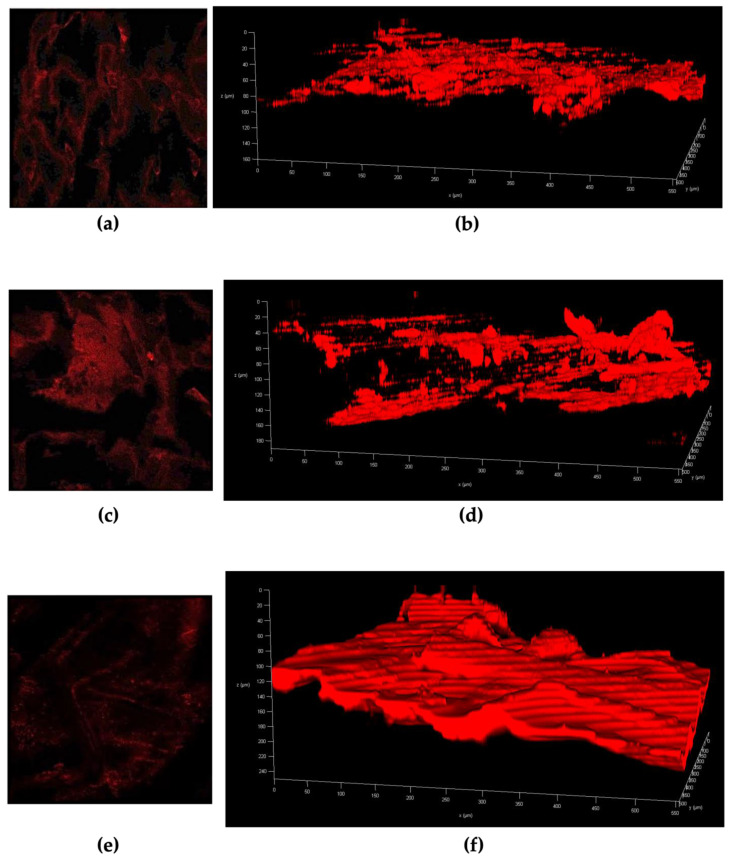
Micrographs of dermatomed skin samples at the end of 24 h permeation study acquired by multiphoton microscopy. (**a**) RB solution (RB-S), stratum corneum; (**b**) RB-S, 3D visualisation; (**c**) RB-loaded aqueous cream BP (RB-C), stratum corneum; (**d**) RB-C, 3D visualisation; (**e**) RB-loaded TF dispersion (RB-TF), stratum corneum; and (**f**) RB-TF, 3D visualisation.

To further evaluate the performance of the RB-loaded dermatological formulations, we investigated RB’s distribution within the dermatomed skin by multiphoton microscopy. Multiphoton microscopy, such as other fluorescence-based microscopical techniques, utilises the intrinsic fluorescence properties of some molecules in order to investigate the delivery of those molecules within the skin’s layers [49]. Herein, we present the micrographs of the skin samples at the end of 24 h of the permeation experiment (Figure 8). Two types of images are shown for each sample (RB-S, RB-C and RB-TF): the first type was acquired as a unidimensional picture of the skin from the top view of the SC, and the second type is a 3D visualisation obtained by multiple horizontal scanning of the dermatomed skin enabling us to see the entire tissue’s thickness. The pictures illustrate only the RB distribution without explicitly showing the skin, which was subtracted by the instrument software.

The overall thickness of the dermatomed porcine skin is 350 µm of which 83.7 ± 16.6 μm of this tissue constitute the epidermis while the SC represents 14.8 ± 4.8 μm. Lastly, the remaining layers of the dermatomed skin are made up of viable epidermis [38]. Regarding RB-S, we hypothesised that the RB preferentially accumulates within the epidermis with minimal-to-no transdermal permeation. Figure 8A shows that RB was visible in the most superficial layers of the skin, and Figure 8B reports an intense yet not entirely homogeneous distribution up to 80–100 μm depth. This depth also reflects the thickness of the epidermis. RB-C was the formulation with the deposition of RB into the skin with very low transdermal permeation. Figure 8C reports a high and non-uniform distribution of RB-C in the outermost skin layer. We previously suggested that the oily phase of RB-C was likely to form a film on the skin surface, acting as a barrier to the permeation of RB loaded in the water phase. Furthermore, considering that the excipients of the oily phase tend to emulsify with the skin components [74], the RB loaded in the oily phase may diffuse through the skin into the dermis and thus result in some degree of, albeit minimal, transdermal permeation. This is corroborated by the data presented in Figure 8D, showing that the RB staining is less consistent than in Figure 8B,F; its distribution was primarily limited in the range of 20–160 μm depth, reaching the dermis. Finally, RB-TF was found to enhance the deposition of RB into the skin and augment the compound’s permeation into the receptor compartment. Figure 8E proves that RB is visible in the most superficial layer, even if the staining is not as intense as in the case of RB-S. On the other side, Figure 8F shows intense and uniform staining, mainly ranging between 40–220 μm depth; in some points, RB is also detectable at the upper 40 μm depth. Hence, RB-TF was primarily deposited on the lower epidermis and dermis, which ultimately resulted in the delivery of the compound into the receiver fluid. This data, in tandem with results from the newly developed HPLC-FD, demonstrates the utility of TF as a novel and potentially versatile dermal and transdermal drug delivery system.

The results discussed here align with the ex vivo permeation and deposition study previously commented on, highlighting the suitability of multiphoton microscopy in investigating drug distribution through the skin. In addition, the imaging capability of this technique elegantly complements the quantitative data presented from the HPLC analysis.

### 3.5. Examination of the Fluorescence Spectra

**Figure 9:**
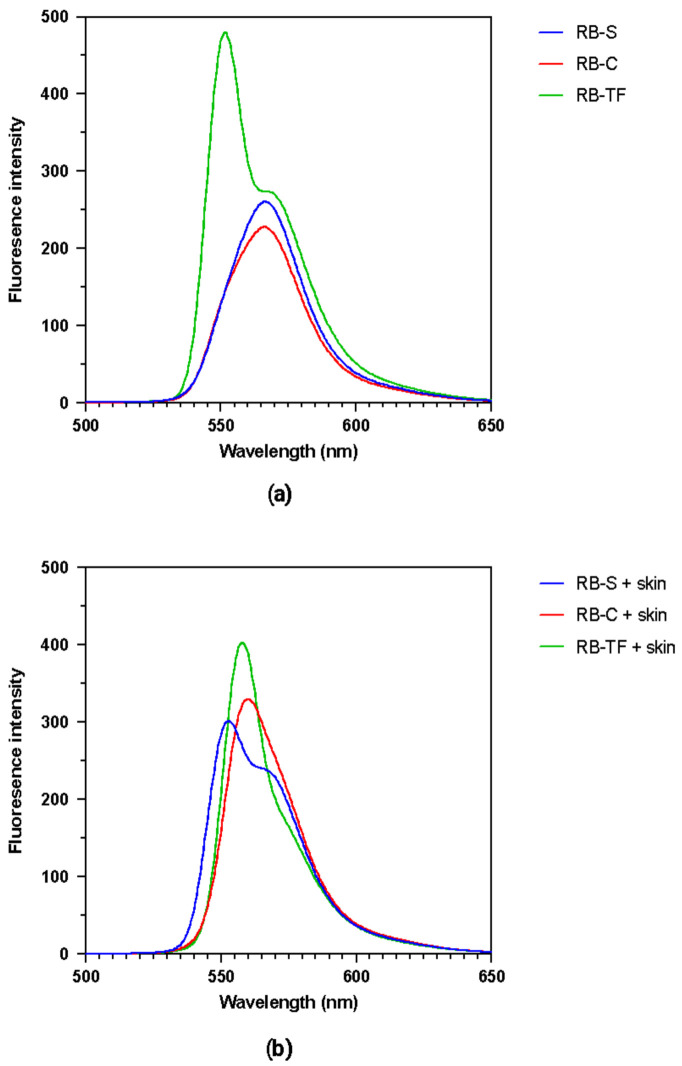
Fluorescence spectra of RB in different environments. (**a**) RB solution (RB-S), RB-loaded aqueous cream BP (RB-C) and RB-TF dispersion. Samples were diluted with PBS, and the auto zero was performed using blank PBS. (**b**) Samples were obtained from skin spiked with RB-S, RB-C and RB-TF. The auto zero was performed using a sample obtained from blank skin.

The investigation of the fluorescence emission spectra of RB was carried out to evaluate RB’s tendency to interact with the components of various environments, e.g., transfersomal dispersion, aqueous cream and skin (Figure 9). The emission spectra recorded highlight how RB’s maximum emission wavelength (λmax) and fluorescence intensity (FI) strongly depend on the composition of the environment in which RB is added. Regarding the spectra of the formulation themselves, RB-S and RB-C showed the same λmax (566.5 nm) and similar FI, 260.1 and 227.4 for RB-S and RB-C, respectively. In the case of RB-TF, the λmax was recorded at 551.5 nm with a FI of 480. The similarity of the RB-S and RB-C spectra can be attributed to the water-based nature of the RB-C. On the other side, RB loading into TF led to a high increase in the FI compared with RB-S and RB-C. The enhanced intensity indicates that RB intercalated through the lipid bilayer mainly in its monomeric form but not as a dimer, effectively limiting the formation of RB aggregates [46]. For this purpose, the aggregation of RB monomers into dimers has been detected in RB aqueous solutions at a concentration above 2.0 × 10^−6^ M, affecting its excitation and emission spectra [80]. Different outcomes were reported concerning skin samples spiked with RB formulations, as the variances in the emission spectra were much less pronounced. In this case, the values recorded were a λmax 552.5 nm and FI 301.8 for RB-S, a λmax 560 nm and FI 329.7 for RB-C and a λmax 588 nm with a FI 403.4 in the case of RB-TF. We previously reported that DMSO dissolves skin lipids and, simultaneously, the lipids constituting the tested formulations [69]. The skin samples, spiked with RB-loaded formulations, were all submitted to the same extraction process described in Section 2.5, which employed DMSO as a lysing solvent. We hypothesised that, in this way, the analysed samples might be similar in composition and molecular bonds, exhibiting similar emission spectra. Nevertheless, a difference can still be noticed since the lipid-based formulation, RB-TF, showed a higher intensity signal.

## 4. Conclusions

The study presented describes the fluorescence-based techniques that might be used to qualitatively and quantitatively determine RB in the skin. Herein, a sensitive analytical method was validated for quantifying RB using the HPLC technique coupled with a fluorescence detector for the first time. The analytical method was validated by considering the standards proposed by the ICH, FDA and EMA guidelines, and it was demonstrated to be specific, precise and accurate. Following topical application, the HPLC method successfully assessed RB’s ex vivo from three different delivery systems. In terms of qualitative determination, it was shown that multiphoton microscopy allows for tracking the RB distribution within the skin tissue, and the analysis of the fluorescence spectra provides crucial complementary information concerning the behaviour of RB under different milieus. Such an approach may play an essential role in preclinical RB formulation screening, representing an effective tool for developing a successful skin-targeted delivery system.

## Figures and Tables

**Table 1:** Chromatographic conditions were used to develop the method for the quantification of RB in the skin.

Condition	Starting Method *	UV-Vis HPLC	FLD-HPLC
Mobile phase	MeOH and buffer pH 8 ^1^ 50/50 *v*/*v*	MeOH and buffer pH 8 ^2^ 60/40 *v*/*v*	MeOH and buffer pH 8 ^2^ 60/40 *v*/*v*
Pump mode	Isocratic	Isocratic	Isocratic
Diluent	Buffer pH 8 ^1^	PBS pH 7.4	PBS pH 7.4
Column	C-18 Chromosil 100-5 μm (250 × 4.6 mm)	C-18 Phenomenex SphereClone^®^ (150 × 4.6 m)	C-18 Phenomenex SphereClone^®^ (150 × 4.6 m)
Column temperature	Ambient	Ambient	Ambient
Excitation wavelength	262 nm	549 nm	556 nm
Emission wavelength	-	-	573 nm
Injection volume	20 μL	40 μL	40 μL
Flow rate	1 mL/min	1 mL/min	1 mL/min
Run time	10 min	7	6
Retention time	2.69 min	3.1 min	4.1 min

* Mannan et al. [40]. ^1^ Potassium phosphate dibasic adjusted with sodium hydroxide or phosphoric acid. ^2^ Ammonium acetate adjusted with sodium hydroxide.

**Table 2:** Properties of the calibration curve were obtained using a fluorescence detector and UV detector to quantify RB with LOD and LOQ values (*n* = 6).

Detector	Slope	Intercept	Linearity ^1^	LOD ^2^	LLOQ ^2^
UV-Vis	190.5	16.84	1.0000	0.60	1.83
FLD	327.3	−58.28	0.9999	0.17	0.54

^1^ Linearity is expressed as R squared. ^2^ LOD and LLOQ are expressed in μg/mL.

**Table 3:** Intra-day and inter-day accuracy and precision of RB (means ± SD, *n* = 6).

	Theor. Concentration (μg/mL)	Exp. Concentration (μg/mL)	Precision (RSD%)	Accuracy (RE%)
Intra-day	8.00	7.89 ± 0.08	1.02	−1.33
	4.00	3.95 ± 0.07	1.91	−1.26
	1.00	0.98 ± 0.03	2.65	−2.42
Inter-day	8.00	7.90 ± 0.08	0.98	−1.17
	4.00	3.93 ± 0.10	2.57	−1.84
	1.00	0.98 ± 0.03	3.06	−1.95

**Table 4:** Dilution integrity developed HPLC method for RB (means ± SD, *n* = 3).

Dilution	Recovery (%)	Precision (RSD%)	Accuracy (RE%)
5 times	104.70 ± 1.02	0.98	4.70
10 times	104.22 ± 1.60	1.53	4.22

**Table 5:** Skin extraction recoveries of RB from dermatomed neonatal porcine skin (means ± SD, *n* = 4).

RB Concentration Added (µg/mL)	ER% ± SD	RSD%
1	97.18 ± 2.33	2.40
4	98.05 ± 8.79	8.96
8	100.82 ± 4.53	4.50

## Data Availability

The data presented in this study are available on request from the corresponding author.
